# Effectiveness of Posttraumatic Growth Interventions among Cancer Survivors: A Systematic Review and Meta-analysis

**DOI:** 10.1192/j.eurpsy.2022.378

**Published:** 2022-09-01

**Authors:** Y.S. Üzar-Özçetin, S. Öcalan

**Affiliations:** Hacettepe University, Psychiatric Nursing, Ankara, Turkey

**Keywords:** pychooncology, posttraumatic growth, mental health, meta-analysis

## Abstract

**Introduction:**

Although cancer is a debilitating experience, it can also increase meaning and satisfaction in one’s life.

**Objectives:**

To investigate the effectiveness of interventions that aim to develop posttraumatic growth among cancer survivors.

**Methods:**

Seven databasis were searched for relevant articles published between 2000 and 2020. The findings of randomized controlled trials related to interventions to effect posttraumatic growth of cancer survivors were included. Hedges’ g and 95% confidence intervals were computed to estimate the effect.

**Figure:**
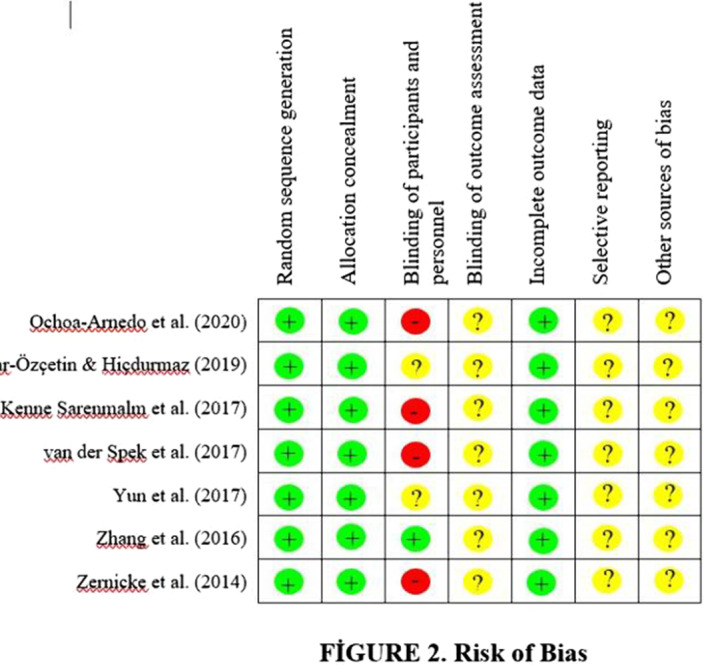

**Results:**

The effect of the interventions on posttraumatic growth among cancer survivors were heterogeneous. The effect size was statistically significant (Tables 1,2).

**Table 1:** 

	k	Total n	Hedges’g	SE	95% CI	z	p	Q Value	**I** ^**2**^	Eggers t	Eggers p
** Overall studies**	t	715	1.761	0.484	[0.812, 2.709]	3.637	< 0.001	182.807	96.718	4.66	.871

**Table 2:** 

				Intervention Effect		
Design	Studies	k	Total n	Hedges’g	SE	95% CI
RCT	Ochoa-Arnedo et al. (2020)	7	140	0.113	0.168	[-0.217, 0.443]
	Üzar-Özçetin & Hiçdurmaz (2019)		76	13.965	1.155	[11.700, 16.229]
	Kenne Sarenmalm et al. (2017)		114	0.423	0.189	[0.053, 0.793]
	van der Spek et al. (2017)		91	-0.161	0.208	[-0.569, 0.247]
	Yun et al. (2017)		174	0.331	0.162	[0.014, 0.648]
	Zhang et al. (2016)		58	2.033	0.321	[1.405, 2.662]
	Zernicke et al. (2014)		62	1.254	0.275	[0.715, 1.793]

**Z = 3.637 P= < 0.001 SE = 0.484 Sd = 1.777**

**Conclusions:**

Posttraumatic growth interventions significantly increased posttraumatic growth among cancer survivors. Health care providers as the main sources of cancer care should be more focused on the achievement of positive outcomes.

**Disclosure:**

No significant relationships.

